# Rare genetic variants influence regional cortical and subcortical grey matter volumes in genetic frontotemporal dementia: A GENFI Study

**DOI:** 10.1002/alz70856_106121

**Published:** 2026-01-08

**Authors:** Saira S. Mirza, Maurice Pasternak, Andrew D. Paterson, Carmela Tartaglia, Sandra E. Black, Sara Mitchell, Morris Freedman, David F. Tang‐Wai, Ekaterina Rogaeva, David M Cash, Martina Bocchetta, John van Swieten, Robert Laforce, Fabrizio Tagliavini, Barbara Borroni, Daniela Galimberti, James B Rowe, Caroline Graff, Elizabeth Finger, Sandro Sorbi, Alexandre Mendonca, Christopher R. Butler, Alexander Gerhard, Raquel Sánchez‐Valle, Fermin Moreno, Matthis Synofzik, Rik Vandenberghe, Simon Ducharme, Johannes Levin, Markus Otto, Isabel Santana, Jonathan D. Rohrer, Mario Masellis

**Affiliations:** ^1^ Hurvitz Brain Sciences Program, Sunnybrook Research Institute, Toronto, ON, Canada; ^2^ Sunnybrook Health Sciences Centre, Toronto, ON, Canada; ^3^ Sunnybrook Research Institute, Toronto, ON, Canada; ^4^ Sandra Black Centre for Brain Resilience and Recovery, Sunnybrook Research Institute, Toronto, ON, Canada; ^5^ LC Campbell Cognitive Neurology Research Unit, Sunnybrook Research Institute, Toronto, ON, Canada; ^6^ The Hospital for Sick Children, Toronto, ON, Canada; ^7^ University of Toronto, Toronto, ON, Canada; ^8^ University Health Network, Toronto, ON, Canada; ^9^ Dr. Sandra E. Black Centre for Brain Resilience and Recovery, LC Campbell Cognitive Neurology, Hurvitz Brain Sciences Program, Sunnybrook Research Institute, University of Toronto, Toronto, ON, Canada; ^10^ Division of Neurology, Department of Medicine, University of Toronto, Toronto, ON, Canada; ^11^ Baycrest Health Sciences, Toronto, ON, Canada; ^12^ Rotman Research Institute at Baycrest, Toronto, ON, Canada; ^13^ Tanz Centre for Research in Neurodegenerative Diseases, University of Toronto, Toronto, ON, Canada; ^14^ Toronto Dementia Research Alliance (TDRA), Toronto, ON, Canada; ^15^ Hawkes Institute, Department of Computer Science, University College London, London, London, United Kingdom; ^16^ Dementia Research Centre, UCL Queen Square Institute of Neurology, University College London, London, London, United Kingdom; ^17^ Department of Neurology and Alzheimer Center, Erasmus Medical Center, Rotterdam, South Holland, Netherlands; ^18^ Research Chair on Primary Progressive Aphasia ‐ Fondation de la famille Lemaire, Quebec, QC, Canada; ^19^ Fondazione Istituto di Ricovero e Cura a Carattere Scientifico, Istituto Neurologica Carlo Besta, Milan, ‐, Italy; ^20^ University of Brescia, Brescia, Lombardy, Italy; ^21^ University of Milan, Milan, MI, Italy; ^22^ University of Cambridge, Cambridge, ‐, United Kingdom; ^23^ Department of Neurobiology, Care Sciences and Society, Center for Alzheimer Research, Division of Neurogeriatrics, Karolinska Institutet, Stockholm, ‐, Sweden; ^24^ University of Western Ontario, London, ON, Canada; ^25^ IRCCS Fondazione Don Carlo Gnocchi, Florence, Florence, Italy; ^26^ Faculty of Medicine, University of Lisbon, Lisbon, Lisbon, Portugal; ^27^ Department of Brain Sciences, Imperial College London, UK, London, London, United Kingdom; ^28^ Division of Neuroscience and Experimental Psychology, Wolfson Molecular Imaging Centre, University of Manchester, Manchester, ‐, United Kingdom; ^29^ Centro de Investigación Biomédica en Red en Enfermedades Neurodegenerativas (CIBERNED), Madrid, ‐, Spain; ^30^ Hospital Universitario Donostia, San Sebastián, ‐, Spain; ^31^ German Center for Neurodegenerative Diseases (DZNE), Tübingen, Germany; ^32^ Laboratory for Cognitive Neurology, Department of Neurosciences, KU Leuven, Leuven, ‐, Belgium; ^33^ Montreal Neurological Institute, McGill University, Montreal, QC, Canada; ^34^ University Hospital, LMU Munich, Munich, Bavaria, Germany; ^35^ University Hospital Ulm, Ulm, ‐, Germany; ^36^ Neurology Department, Centro Hospitalar e Universitário de Coimbra, Coimbra, ‐, Portugal; ^37^ Dementia Research Centre, Queen Square Institute of Neurology, University College London, London, ‐, United Kingdom; ^38^ Dr. Sandra Black Centre for Brain Resilience & Recovery, Sunnybrook Research Institute, Toronto, ON, Canada

## Abstract

**Background:**

There is substantial heterogeneity in clinical presentation of genetic Frontotemporal Dementia (FTD), even within the same family. This suggests that additional heritability may exist and contribute to this variable presentation. We examined whether gene‐based aggregate burden of genome‐wide rare variants (minor allele frequency [MAF]: ≤1%) contribute to variation in regional cortical and subcortical grey matter volumes, after controlling for effects of causative mutations in *GRN*, *MAPT*, and *C9orf72*.

**Method:**

This study was embedded within the GENetic Frontotemporal dementia Initiative (GENFI), which recruits genetic FTD cases and their asymptomatic at‐risk family members, both carriers and non‐carriers of FTD mutations. We included 518 participants with genotype (Neurochip; imputed against TOPMed), and T1w‐MRI brain volumetric data. Gene‐based burden tests that aggregate the number of rare variants by gene were used to examine the association of rare variants (MAF: ≤1%) with regional cortical and subcortical grey matter volumes (70 regions of interest [ROIs]), controlling for age, sex, total intracranial volume, mutation status, scanner site, population stratification, and family membership (kinship matrix) using RVTests. Annotations for loss of function mutations (LOF): start gain, stop loss, start loss, essential splice site, stop gain, normal splice site, and non‐synonymous. Multiple testing correction accounted for the number of genes and number of independent grey matter volumes as calculated by matSpD (*p*‐value threshold: 0.05/(17,053x42) = **6.98 x10^‐8^)**.

**Result:**

Aggregate burden of LOF mutations (*DNAJB8‐AS1, WDR26, RDM1P5, BSND, CNOT2, DDA1, ASAH2B, PPM1A, HOXD13, ALDH1A1, CENATAC, ANKRD45)* was associated with significantly lower volumes within the left temporal lobe (ROIs: left temporal and lateral temporal left), and greater volume in the putamen bilaterally (*TSACC)*. All genes are protein coding, except the *DNAJB8‐AS1* (antisense RNA) and RDM1P5 (pseudogene), and are variably expressed in the brain. Molecular functions of significant genes involve regulation of gene expression, transcription, and cell cycle, ion channel function, and chromosomal segregation. *WDR26* and *CNOT2* genes have been implicated in neurodevelopment and neurological disorders respectively; *BSND* gene is involved in neurotransmission.

**Conclusion:**

Identification of deleterious or protective rare variants contributing to FTD imaging phenotypes may help identify genetic modifiers of familial FTD. Replication in larger cohorts is needed.

